# AI-Powered Transformation of Healthcare: Enhancing Patient Safety Through AI Interventions with the Mediating Role of Operational Efficiency and Moderating Role of Digital Competence—Insights from the Gulf Cooperation Council Region

**DOI:** 10.3390/healthcare13060614

**Published:** 2025-03-12

**Authors:** Fatema Saleh AlDhaen

**Affiliations:** Department of Management, Marketing and Information Systems, College of Business & Finance, Ahlia University Manama, Manama 10878, Bahrain; faldhaen@ahlia.edu.bh

**Keywords:** AI intervention, digital competence, operational efficiency, patient safety, healthcare, GCC region

## Abstract

**Background/Objectives:** The purpose of this study is to investigate the role of the adoption of artificial intelligence technology in improving patient safety in hospitals working in gulf Cooperation Council (GCC) countries, with a focus on the mediating role of operational efficiency and moderating effect of digital competence. **Methods:** Applying a quantitative, cross-sectional, and explanatory research design, data were gathered from 300 healthcare professionals across five hospitals in the GCC region. **Results**: The results show that AI interventions improve patient safety by improving operational efficiency, while the digital competence of healthcare professionals further enhances the effectiveness of AI interventions. The findings exhibit that AI interventions enhance patient safety through high diagnostic accuracy at 95.2%, combined with 1.8% low medication errors and 92.4% efficient timely interventions. Based on previous research, the proposed approach achieves 5.7% better diagnostic accuracy and 1.4% fewer medication errors, together with 4.9% enhanced timely interventions. **Conclusions and Implications:** These findings highlight the importance of adopting AI technologies and enhancing digital competence among healthcare professionals to optimize operational efficiency and ensure safer healthcare delivery. This study offers actionable insights for healthcare managers and policymakers, emphasizing the need for AI-driven training programs and infrastructure investments.

## 1. Introduction

Artificial intelligence (AI)-powered interventions in the healthcare sector are changing the ways through which the systems deliver medical services by ensuring the improvement of safety, functionality, and quality of services [1]. Universal measures involve the protection of patient health, including diagnostic safety, safe medication practices, patients’ vital signs’ real-time tracking, and timely notification about important interventions, among others [2]. Even though the technological development is growing very quickly, patient safety and the optimal management of healthcare resources in the healthcare sector, especially in the GCC countries, remain a challenge. This is compounded by the fact that the region has a very young population, the occurrences of diseases such as diabetes and other chronic diseases are on the rise, and many healthcare facilities depend on an expatriate workforce [3]. It is for this reason that AI interventions have come out as great innovative solutions that can handle the challenges by enhancing efficiency, helping make clinical decisions, and optimizing resource use. There have also been improvements to clinical operations where machine learning algorithms and predictive analytics, as well as automated systems, have been used by AI [4]. However, to achieve the realization of these aspects of AI technology, the digital competence of healthcare providers and the result obtained from the real factor of AI must also be considered. Healthcare digital literacy means the ability of healthcare professionals to adequately harness digital tools and platforms and is an important determinant of AI appropriateness. They postulate that the healthcare organization with more developed digital literacy can trust, adopt, and effectively implement AI tools [5]. Consequently, organizational performance improves and results in decreased incidences of labor-intensive protocols, the prescription of common protocols, and smooth coordination amongst the working healthcare teams. Enhanced operational efficiency plays a moderating role in patient safety in that it allows for timely identification as well as intervention, the correct identification of aetiology, and the prevention of adverse events.

The adoption of AI technologies to transform healthcare delivery particularly in solving patient safety concerns has achieved greater success. For example, Zhao, Zhang [6] indicated the role of AI systems in decreasing diabetes-related avoidable hospitalizations through enhanced primary healthcare resourcing. In the same vein, Luo, Ahmad [7] highlight the importance of perceived ease of use, usefulness, and financial strength in implementing health information systems (HIS) with hospital size as the moderator. The research highlights the potential of AI to enhance healthcare-related outcomes, particularly in developing countries like those in the GCC region.

In the case of the GCC region, particularly where most of the countries have embarked in launching ambitious healthcare reforms and have invested a good deal of resources in technology [8], the use of AI interventions as previously highlighted has offered a window of opportunity in addressing some of the causes and effects of patient safety incidents. For instance, through the use of real-time monitoring systems in conjunction with AI, it becomes easy to alert important conditions such as sepsis or cardiac arrest in a timely manner to help in responding appropriately [9]. Likewise, in rating clinical patterns and anomalies, it is possible to minimize the margin of statistical error with the help of AI-based technologies. However, there are also several problems that impede AI implementation in the healthcare systems of the GCC countries, including digital preparedness, digital infrastructure, and workers’ ability to innovate and adopt novel technologies [10]. It is critical to know how each of the components impacted patient safety in this region in order to construct key strategies for increasing the benefits from AI interventions, digital competencies, and operation effectiveness.

Thus, the present study intends to explore the use of AI interventions to maintain patient safety across the selected countries of the GCC and the mediating role of operational efficiency and moderating influence of digital competence. Therefore, through the analysis of these vectors, this research aims at identifying how AI technologies can be used in the provision of healthcare in order to support patient care needs that might be unique to certain geographic areas.

Expatriate healthcare professionals deliver most of the healthcare services across the majority of GCC nations. Included in the challenges that the healthcare industry across the GCC region faces is the dependence on expatriate healthcare professionals [11]. The United Arab Emirates [85%] and Saudi Arabia [78%] have the highest percentage of expatriate healthcare staff in the GCC region [12,13]. In the UAE, local nurses constitute 3% of the 24,000–25,000 nurses working in the country [13]. In the same vein, the ratio of expatriate nurses across the GCC region is the highest among all healthcare professionals [3]. The high cost and high turnover of expatriate healthcare professionals create financial and uncertainty problems for the industry. It is expected that AI integration will considerably reduce the dependence on expatriate healthcare professionals.

The GCC region experiences growing chronic disease prevalence of diabetes and cardiovascular conditions, which requires intelligent AI systems to boost patient treatment quality and management resource utilization [3]. The medical establishments based on expatriate medical staff leads to unequal digital capability, which defines the pace of AI technology implementation and its performance success [14]. Healthcare reforms, such as Vision 2030 in Saudi Arabia and AI Strategy 2031 in the UAE, support AI adoption yet encounter obstacles from infrastructure gaps and resistance to digital transformation [10]. The variable technological development levels among GCC nations affect AI implementation because these countries need to invest in enhancing digital infrastructure [9]. Safety protocols combined with ethical requirements about AI transparency must be followed for implementing responsible AI solutions. Standardized policies help minimize potential risks while making AI healthcare transformation more possible [15].

Despite significant differences in organizational settings of the GCC countries, the healthcare systems in the region share several structural commonalities, particularly in their strategic moves on AI adoption, digital transformation, and operational efficiency improvements [16]. The national healthcare strategies, such as Vision 2030 of Saudi Arabia, Strategy 2031 of the UAE, and The Supreme Council of Health of Qatar, present a unified regional commitment to modernize healthcare services through digital and AI technologies [17]. The GCC healthcare industry profoundly relies on an expatriate workforce. Healthcare professionals including physicians, surgeons, nurses, paramedics, and administrative personnel are largely hired from the international labor market [18]. The GCC countries largely operate under a closely aligned legal and ethical framework governing healthcare services delivery. The GCC nations have comparable healthcare financing models, cultural norms, and economic structures that influence the delivery of healthcare services. Government-subsidized healthcare services, public–private healthcare partnership, and insurance-based patient care models are prevalent across the region. All these characteristics reduce potential variation in healthcare delivery systems in the GCC world.

In this research, the researcher endeavors to add to the existing literature on AI in the health system and provide possible policy implications and implementation strategies to policymakers and healthcare managers in the GCC countries. However, a gap has been observed in experimental and quantitative studies about how, and through what process, AI offers patient safety in healthcare, even with an increasing number of works published on the use of AI in healthcare in recent years, especially in the context of the Gulf Cooperation Council (GCC) countries. AI has been the subject of extensive research in terms of technical AI, or the global changes it is likely to bring to healthcare, but little attention was paid to how factors may influence the efficiency of AI on patient outcomes in the region by the digital competence of healthcare workers and the efficiency of the delivered healthcare services. However, in studies, this gets lost especially because the challenges that the GCC region presents may be different from other regions, such as healthcare workforces and varying digital readiness to implement technologies and adjoining work in developing and changing healthcare structures. These gaps in regional research make it difficult for policymakers and healthcare administrators in the GCC to make effective decisions on the use of AI and its impact on patient safety. This work fills this void by exploring the relationship between AI interventions, digital competencies, effectiveness, and safety in the GCC countries. Thus, by highlighting these interconnected variables, this research offers a holistic view of the way AI can be adopted to improve patient safety, in a context of increasing technology adoption and heterogeneity of care needs in the region of interest. In addition, this study also extends the current knowledge on AI implementation by establishing the mediating role of operational efficiency and the moderating role of digital competence. In doing so, this study is informative to the literature focusing on the use of AI in the delivery of health services, as well as useful to policy makers and practitioners in the GCC to understand and manage the potential opportunity and risks of using AI in the context of the healthcare services in the region.

## 2. Literature Review

Information technology, particularly artificial intelligence (AI), has become the solution to many of the challenges affecting patient safety, including diagnostic mistakes, delayed treatments, and system management drawbacks. Clinical Decision Support Systems (CDSS) and predictive analytics are two emerging applications of AI that have revealed potential in increasing diagnostic reliability and optimizing actions [14,19]. For instance, in the diagnosis process, the AI systems that analyze big data sets have provided higher accuracy in the diagnosis of diseases, even complex ones like cancers and cardiovascular diseases, and, thereby, little chances of wrong diagnosis [20]. Real-time applications with the backing of AI have empowered further constant surveillance in the monitors of patient status and early alert in cases such as sepsis and other cardiac arrest [21]. These tools help save time that is required in delivering medical responses, which are essential in averting adverse impacts. However, there is still much to overcome, including the problems of how to merge AI into current healthcare structures, and how to ensure clinicians trust the systems [22]. One of the prerequisites of a positive adoption of AI technologies is the digital competence of the healthcare workers. Higher digital competency makes physicians employ AI tools and rely on their suggestions that benefit patients [23]. On the other hand, digital incompetency may lead to resistance to the use of AI, leading to the negation of its benefits [24]. The implementation of AI technologies is being extensively studied with particular emphasis on its role in improving operational efficiency and patient safety. Zhao, Zhang [6] explain how AI systems can minimize avoidable hospitalizations by optimizing resource allocation and enhancing diagnostic accuracy. Luo, Ahmad [7] further underline the impact of digital literacy and organizational factors in the effective integration of AI technologies.

In the context of the GCC, where healthcare systems are relatively developing, digital competencies among the healthcare providers are moderate. To this end, the need for customized training interventions and digitization upskill programs to explain AI-supported applications are needed [25]. Research has pointed to the benefits of building clinical AI trust to improve clinicians’ comfort using AI tools, especially in tight-knit corners, such as in the intensive care unit or diagnostics [15]. There is also evidence of the mediating role of operational efficiency in the context of AI interventions in healthcare settings; it involves an ability to enhance healthcare performance and reduce the utilization of resources in order to augment patient safety [26,27]. AI has helped to alleviate lengthy paperwork, make clinical work adoptable, and enhance interdisciplinary company [28,29]. For example, Lekadir, Feragen [30] noted that the application of automated medication administration reduced errors involving drug-interaction and dosing calculations.

Nevertheless, how operating efficiency moderates the effects of AI interventions on patient safety has not been well investigated, especially in GCC countries. Filling this gap could help in understanding how AI technologies can solve regional problems including the shortage of human resources and high patient-to-nurse ratio [31]. AI interventions have a direct impact on the key healthcare goal of ensuring patient safety. These include reducing diagnostic mistakes, avoiding adverse drug events, and offering early notification of life-threatening pathophysiological states [32]. It has been found that AI-based tools improve the specificity of the diagnostic process due to the possibility of pointing out the patterns that were not considered by the clinician, for example when analyzing imaging tests or laboratory results [33]. Indeed, in the GCC context where healthcare systems are escaping the development phase to attain higher quality benchmarks, the capacity for AI to make patient safety is enormous. For example, AI enhanced continuous vital sign measurement has demonstrated effectiveness in reducing critical events for high-risk patients [8,34]. Nevertheless, its efficiency highly depends on the lack of infrastructural and training impediments to AI technologies’ integration [35].

## 3. Theoretical Framework

Short of this, there is a wealth of theory available that could be underpinning this work. However, to fill this gap, this study will hold the following theories to lend theoretical underpinning and to explain the hypothesized relationships. The Technology Acceptance Model (TAM) is a theory that dichotomously explains the adoption and usage of technology by users as advanced by Davis [36]. TAM posits that two primary factors influence technology adoption: acceptance behavior including perceived usefulness (PU) and the perceived ease of use (PEOU). In the context of this study, the Technology Acceptance Model (TAM) supports the connection between the AI initiatives applied and their usage by the HCPs depending on their levels of digital competencies. It has been found that if the healthcare providers regard AI as relevant and easy to implement, they will adopt it in practice, which, in turn, will promote the efficiency of the system and reduce risks for the patients. The Resource-Based View Theory (RBV) [37] pays attention to resources as key to achieving competitive advantages, and it includes technology, knowledge, or skill. As a form of flow, operational efficiency is the resource that is improved through AI intercessions in this research. The RBV framework connects the use of AI tools (technological resources) to the enhancement of the business operation of healthcare organizations and consequently better outcomes of patient safety. Systems Theory [38] is centered on interactions, reliance, and dependency of the numerous elements present within an organization. Therefore, this research work embraces a systems perspective to comprehend how the uses of AI interventions, digital competency, operational productivity, and patient safety are interconnected in the health system. The theory will back the notion that proper functioning of one element (for instance, operational efficiency) through AI changes will result in better system performance, for example, patient safety.

### 3.1. Definition of Variables

1. **AI Interventions (Independent Variable)**

It refers to the application of AI technologies, systems, or algorithms to support, enhance, or replace healthcare processes, tasks, and decision making [39]. AI intervention is the use of artificial intelligence technologies, like machine learning models, real-time monitoring systems, automated decision support tools, and predictive analytics. The AI intervention enables healthcare professionals to perform better, by enhancing patient safety, reducing diagnostic errors, and optimizing workflow efficiency.

2. **Digital Competence (Moderator)**

The ability of healthcare personnel to effectively use digital tools and AI technologies [40].

3. **Operational Efficiency (Mediator)**

The optimization of workflows, reduction of errors, and efficient resource utilization within healthcare organizations [41]. The operational efficiency metrics evaluate time taken to complete a procedure, reduction in diagnostic and treatment errors, coordination and procedural adherence, patient throughput, and allocation medical resources.

4. **Patient Safety (Dependent Variable)**

The prevention of errors and adverse outcomes, including improved diagnostics, reduced medication errors, and timely interventions [42].

### 3.2. Hypotheses

1. **H1: The AI interventions in healthcare organizations are likely to enhance operational efficiency.**

Digital and artificial intelligence technologies reduce time-intensive manual tasks, standardize workflows, automate processes, and lead to greater operational efficiency. For example, predictive analytics minimizes diagnostic delays, and automated medication systems reduce prescription errors [43].

2. **H2: Operational efficiency within healthcare organizations is likely to augment patient safety.**

Streamlined workflows and reduced errors ensure that healthcare providers can focus on patient care, leading to improved safety outcomes. Enhanced efficiency allows timely interventions and reduces the likelihood of adverse events [21].

3. **H3: Operational efficiency mediates the relationship between AI interventions and patient safety.**

AI-powered interventions enhance operational efficiency, which in turn contributes to better patient safety outcomes. The mediating effect is crucial in understanding how AI tools indirectly impact safety through process improvements [44].

4. **H4: Digital competence moderates the relationship between AI interventions and operational efficiency, such that the relationship is stronger when digital competence is high.**

The proper use of AI technologies depends on the digital skills of healthcare professionals. Higher digital competence ensures better integration and utilization of AI technologies, enhancing operational efficiency [45].

5. **H5: Introduction of AI-powered interventions in healthcare organizations are likely to enhance patient safety**

The application of digital and AI-powered technologies in healthcare can enhance patient safety by improving the accuracy and efficiency of clinical decision-making. Based on medical data, AI systems identify patterns and extend meaningful recommendations to overcome medical and diagnostic errors. It is through predictive analytics and continuous monitoring that early signals of deterioration are identified to make appropriate changes.

## 4. Methodology

### 4.1. Research Design

This study used a quantitative, cross-sectional, and explanatory research approach to analyze the correlation between AI interventions, healthcare organization operational efficiency, individual digital competencies of the employees, and patient safety in healthcare organizations in the GCC countries. The use of a quantitative method was considered suitable for the analysis of the cause–effect relationships between variables, while the cross-sectional method provided an opportunity to collect data at a given time only to reveal the state of AI implementation and its results. The explanatory design was concerned with explaining how AI interventions impacted patient safety in terms of process effectiveness and organizational digital literacy. This study integrated both subjective self-reported data and objective clinical performance metrics to evaluate the impact of AI adoption on patient safety and operational efficiency. While the subjective data were collected via structured questionnaires, objective measures were obtained from hospitals’ databases. To develop objective performance metrics, data on the chosen indicators were collected for both the pre-AI and post-AI implementation periods.

### 4.2. Population and Sample

- **Population:**

The target population consisted of healthcare professionals, including physicians, nurses, and administrative staff, working in hospitals and clinics in the GCC region where AI technologies were implemented. These professionals were directly or indirectly involved in decision-making, patient care, and operational processes supported by AI systems.

- **Sample Size:**

Based on the requirements for Structural Equation Modeling (SEM) using SmartPLS, a minimum sample size of 10 respondents per item in the measurement scale was recommended [46]. With an estimated 30 items across the study variables, the target sample size was set at 300 respondents to ensure sufficient statistical power for hypothesis testing. Close ended questionnaires were administered to a randomly selected sample of 350, of which 318 were received back. 300 questionnaires complete from each respect were selected for analysis. In addition to these questionnaires, objective data on hospital performance indicators were extracted from the institutional databases of each hospital.

- **Homogeneity of Sample:**

The selection of sample hospitals across various GCC countries was based on their alignment with key AI adoption and digital transformation initiatives in the respective countries. Despite being distinct countries, the healthcare systems share fundamental commonalities, including the large number of expatriate healthcare professionals, standardized healthcare policies, and similar degree of AI readiness. Additionally, regulatory frameworks administering AI adoption and digital competency trainings in these settings follow coherent regional guidelines ensuring AI-powered healthcare interventions operate under comparable conditions. These similar characteristics justify treating the selected hospitals as a homogeneous sample for the study in hand.

- **Exclusion Criteria:**

A total of 18 questionnaires were excluded due to incomplete or inconsistent responses to maintain data integrity and a robust analysis. Nine questionnaires were excluded from administrative staff; six from nurses and three from doctors were discarded because they were missing responses exceeding 20% of the total survey items or exhibiting response pattern indicative random or non-engaged response (e.g., selecting the same response options for all the questions). Twelve questionnaires were excluded due to missing responses to items, four questionnaires were discarded due to inconsistent response pattern, and two questionnaires were eliminated due to the absence of demographic information. This exclusion criterion was followed to minimize measurement errors arising due to low-quality responses and enhance the reliability and validity of dataset.

- **Sampling Technique:**

The sample was drawn from five tertiary hospitals, each located in different GCC regions. A random sampling technique was applied for sample selection. Physicians, nurses, and administrative staff were included in the sample. This method minimized selection bias and enhanced the generalizability of the findings.

### 4.3. Measurement Scales

The constructs were measured using a 5-point Likert scale (1 = Strongly Disagree, 5 = Strongly Agree), with items adapted from previous studies to ensure validity and reliability (for complete questionnaire see Appendix A). The English version of the questionnaire was used, as the respondents, being educated, had no difficulty understanding English. Therefore, translation into another language was deemed unnecessary. AI intervention was measured with the Digital Maturity Assessment (DMA) implemented by the NHS. The questions focused on the use of predictive analytics, real-time monitoring, and decision support systems. For example, “The healthcare facility utilizes predictive analytics for patient diagnosis”, “The AI system in our hospital provides real-time alerts for critical patient conditions”, and “Healthcare professionals often use AI tools to support clinical decision-making”. This scale has been extensively used by researchers to measure the extent and effectiveness of AI systems implemented for diagnostic support, real-time monitoring, decision-making, and workflow automation in healthcare settings [47,48]. Operational efficiency in healthcare was measured with the scale used by Moshiri, Aljunid [49]. The scale measured task completion times, error rates, and workflow optimization. Questions included “The system effectively assists in reducing task duplication”,” The system contributes to faster decision-making in our hospital in the process of provision of care”, and “operational bottlenecks are usually addressed with AI interventions”. Digital Competence was measured with the 12-item scale developed and used by Schwarz, Bieg [50]. The items included “I can use the digital devices used in our hospital without difficulty”, ”I can organize and store digital information efficiently”, and “I can adapt to new digital tools and technologies quickly”. Patient safety was measured through the Hospital Survey on Patient Safety Culture (HSOPSC) [51]; however, certain changes were made to make it more aligned with the given context. The questions included “healthcare professionals actively doing things to improve patient safety”, “Hospital management provides a work climate that promotes patient safety”, and “Staff will freely speak up if they see something that may negatively affect patient care”.

To establish objective hospital performance metrics, the selected indicators included a decrease in medication error rate, a reduction in patient wait times, and an improvement in diagnostic accuracy. The comparative benchmarks shown in Table 9 were extracted from a review of prior studies that examined patient safety outcomes under non AI-supported conditions. The referenced literature [4,9,14] provides empirical evidence on medication errors, diagnostic accuracy, and timely interventions where AI tools were not used or at the early stages of AI implementation. Data from these sources were extracted and normalized where deemed necessary, and comparted with the AI-powered approaches that implemented AI technologies such as predictive analytics, real-time monitoring, and automated decision support systems.

### 4.4. Pilot Study

A pilot study was carried out prior to the full-scale data collection to ensure the reliability and clarity of questions. The pilot test included 31 respondents from a hospital outside the sample frame to ensure representativeness without overlapping with the main sample. The outcome of the analysis depicted that the survey questions were clearly understood and pertinent to the context. Cronbach alpha values for all the variables were above 0.07, confirming internal consistency. Minor revisions were made to some questions for clarity based on the feedback received. So, the questionnaire was validated prior to administration to ensure reliability and relevance to the objectives.

### 4.5. Data Analysis Method

Data were analyzed using SmartPLS, a Partial Least Squares Structural Equation Modeling (PLS-SEM) tool, due to its suitability for testing complex relationships and predictive models. The analysis process included the following steps. Internal consistency was assessed using Cronbach’s Alpha and Composite Reliability (CR). A reliability value of 0.7 or above was considered acceptable [52]. The Average Variance Extracted (AVE) was calculated to determine the extent to which the indicators reflected their respective constructs. An AVE of 0.5 or higher was considered satisfactory [52]. The Fornell–Larcker Criterion and the Heterotrait–Monotrait (HTMT) Ratio were used to assess discriminant validity, ensuring that constructs were distinct from one another. HTMT values below 0.85 were deemed acceptable. The Variance Inflation Factor (VIF) was calculated to detect multicollinearity [53]. VIF values below 5.0 indicated no significant collinearity issues among variables [54]. The Standardized Root Mean Square Residual (SRMR) was used to evaluate the model fit, with values below 0.08 indicating good fit. The R^2^ value measured the variance explained by the independent variables in the dependent variable. To test the hypotheses, Path coefficients and *p*-values were obtained using the bootstrapping technique with 5000 subsamples. Hypotheses were supported if *p*-values were less than 0.05 and the path coefficients were significant.

### 4.6. Ethical Considerations

This study adhered to ethical guidelines for research involving human participants. This study observed ethical standards outlined in the Helsinki Declaration. The respondents participated voluntarily, with informed consent obtained prior to data collection. A written consent form was created containing the goals, procedure, confidentiality, voluntary participation, potential benefits of the study, and their right to withdraw at any stage without penalty. The respondents were administered with the questionnaire after they expressed their willingness to participate in the study. The confidentiality and anonymity of all the participants were strictly maintained. An ethical approval letter (No. BF-2024-2025-12) was obtained from the ethics committee of Ahlia University Bahrain.

## 5. Data Analysis

The data analysis for this study consists of descriptive statistics, demographic profiling, and hypothesis testing using Partial Least Squares Structural Equation Modeling (PLS-SEM) with SmartPLS. The analysis includes reliability testing, convergent and discriminant validity, multicollinearity checks, R^2^ values, and path analysis for hypothesis testing.

### 5.1. Descriptive Statistics and Demographics

Demographic data of the respondents reflected a diverse sample in the study (Table 1). The participants were 300 healthcare professionals from the GCC countries, of which 60% were male and 40% were female. The largest portion of participants was 31–40 years old (46.7%); the participants aged 20–30 years were the second largest group (33.3%); and the remaining participants were older than 31 years. If categorized on the basis of occupational status, 40% of the respondents were physicians, 33.3% were nurses, and 26.7% were administrative workers. In terms of healthcare experience, the majority had a work experience of between 5–10 years (40.0%) and a smaller proportion had worked in healthcare for over 20 years (13.3%). It can therefore be seen from this distribution that the samples drawn were drawn from different folds of age, gender, professional designation, and experience, thereby making the lateral study more reliable.

### 5.2. Reliability and Convergent Validity

In order to test the internal consistency and reliability of the constructs, values of Cronbach Alpha and variable Composite Reliability were computed (Table 2). Concerning the reliability of the questionnaire and each of the constructs explored, AI interventions, operational efficiency, digital competence, and patient safety presented Cronbach’s Alpha values of more than 0.7 and CR of over 0.8. To test for convergent validity, we applied the Average Variance Extracted (AVE), which in all the constructs was above 0.5. These overall outcomes strongly support the reliability of the indicators adopted for each of the reflective constructs.

### 5.3. Discriminant Validity

Discriminant validity was assessed using both the Fornell–Larcker criterion and the Heterotrait–Monotrait (HTMT) ratio. The Fornell–Larcker results showed that the square root of the AVE for each construct was greater than its correlations with other constructs, satisfying the discriminant validity criteria (Table 3). Additionally, the HTMT ratio values for all the constructs were below 0.85, further confirming that the constructs were distinct from one another (Table 4).

### 5.4. Multicollinearity and Model Fit

To check multicollinearity, Variance Inflation Factor (VIF) statistics were computed for all the constructs. The VIF ranged between 1.95 to 2.25; the cut-off value is 5, thus, one cannot have a multicollinearity problem (Table 5). The goodness of fit was assessed by Standardized Root Mean Square Residual (SRMR) (Table 6), the value of which was 0.061, and since it finally lies less than 0.08, the fit is considered robust. Furthermore, the operational efficiency and patient safety model yielded satisfactory R^2^ values of 0.41 and 0.46, respectively, thus indicating that substantial variance in these constructs was explained by the independent variables. Both R^2^ values were above the cut-off of 0.26, showing the capability of the model as a good predictor.

### 5.5. Structural Model and Hypothesis Testing

Table 7 provides an understanding of the relative strength of the relationships between the path coefficients between the structural model tested hypotheses. All the hypotheses postulated were tested statistically using path coefficients (β), T-statistics, and *p*-values obtained from the bootstrapping method with a sub sample of 5000, to enhance the validity of the results. This study showed that AI interventions had a positive association with operational efficiency, and this was statistically significant (standardized coefficient = 0.48; t = 7.89; *p* = 0.001). Supporting Hypothesis 1, this outcome demonstrates that AI interventions make a substantial improvement in operational efficiency by cutting cycle times, eliminating human mistakes, and automating tedious tasks. These results show that the path coefficient of this effect is significant and large, underlining the importance of AI technologies for healthcare management. Thus, operational efficiency revealed a substantial positive relationship with patients’ safety (r = 0.52, t = 8.34, *p* = 0.001), which confirms Hypothesis 2. This finding supports the concept that operational efficiency helps drive safer healthcare through a proximal relationship, as better processes ultimately translate to fewer mistakes and a more effective ability to respond in real time. Positive significance was also established between IT interventions and patient safety with the beta coefficient (β = 0.34, T = 6.12, *p* = 0.002), confirming Hypothesis 3. It is therefore clear that, while the direct effect was moderate, AI has a twofold effect on patient safety through the stronger mediated indirect effect through operational efficiency. Consequently, this specific study stresses the need for a balance between changes made directly by artificial intelligence systems and process-based modifications for ideal safety performance. As for the moderator, the research revealed that digital competence can become an important moderator of the model. H4 was supported, as the cross product between the digital competence and the AI interventions significantly and positively affected operating efficiency (β = 0.39, T = 5.76, *p* = 0.001). This result suggests that digital competence is higher for the corresponding interventions among HCPs, and this endorses the notion that enhanced efficiency by the use of the AI tools enhances the operations performance accelerators. The data analysis supported Hypothesis 5, demonstrating a direct relationship between AI Intervention and patient safety. Although the statistical evidence was significant (β = 0.19, T = 3.98, *p* = 0.002), the relationship was not that strong. This weak relationship suggests the existence of a partial mediation of operational efficiency between the relationship of AI intervention and patient safety.

Moreover, all of the proposed hypotheses are supported in the overall path analysis of the theoretical model. The analyzed results indicate the fact that the constructs are logical and consistent; AI interventions, conditionally mediated by operational efficiency, contribute positively to the improvement of organization performance, which ultimately raises the levels of patient safety. By rendering support for the proposed model, these findings assure a higher empirical generalizability, and thereby further stress the need of developing and enhancing digital competence as well as achieving operational efficiency to enhance the effectiveness of AI in healthcare organizations.

### 5.6. Clinical Performance Metrics

The objective clinical performance data (as shown in Table 8) align with the statistical findings presented. Specifically, the implementation of AI led to a 1.5–2.0% reduction in treatment errors across the five selected hospitals, demonstrating a considerable improvement in patient safety. Additionally, patient wait times decreased by 13–17 min, enhancing overall efficiency in healthcare delivery. Furthermore, AI adoption contributed to a 5–7% increase in diagnostic accuracy, reinforcing its role in improving clinical decision-making. Importantly, the frequency of AI usage surged by 68–74%, indicating widespread integration, digital competence of employees, and acceptance of AI-assisted tools in the selected hospitals.

## 6. Discussion

The results are highly consistent with the expected relationships and thus lend significant support for the research hypotheses, as well as advancing the knowledge on AI’s potential to enhance the delivery of and progress in healthcare. The implications of the results are examined with regard to the developed hypotheses based on the identified opportunities and gaps in the literature, as well as with an aim to provide practical recommendations for the healthcare industry. Preliminary expectations of the first hypothesis, which posited that the use of AI interventions would have a positive relationship with operational efficiency, were confirmed (β = 0.48, *p* < 0.05). This result supports other research on how the application of AI technologies in healthcare organizations improves efficiency and reduces task complexity and errors [14,21]. For example, analytics of care episodes and diagnostic tools have been demonstrated to improve process optimization because they free up the time of care professionals to focus on the important aspects of patient care. This result supports the importance of adopting AI as a means to enhance resource productivity and efficiency improvement and to augment operational capacities in the specific setting of GCC healthcare systems that are struggling to overcome problems with the shortage of human resources and increasing patient loads. The analysis also revealed that operational efficiency had another positive correlation with the results obtained for patient safety (Hypothesis 2) with a high path coefficient (β = 0.52, *p* = 0.000). This is in line with previous research that shows that resourceful healthcare systems provide greater capacity to ensure timely interventions, accurate diagnosis, and fewer medical mistakes [55,56]. Thus, increased operational efficiency reduces the time span and disorganization of healthcare teams, which hold a significant role in safety results. These results support Hypothesis 3, which postulated that operational efficiency partially mediates the relationship between AI interventions and patient safety. Thus, it is possible to consider two paths: increasing the usage of AI technologies in production and improving the format of work organization, in which integrated AI technologies are used safely and efficiently. The path between the use of AI interventions and improved patient safety, which was proposed in Hypothesis 3, was also confirmed (β = 0.34, *p* = 0.001), although with a moderate strength. Although the direct utilization of AI tools contributes towards enhanced safety in decision making, continuous monitoring, and predictive rationality, the contingency effect of increasing operational effectiveness seems to yield a better positive impact. This differentiation is in accordance with previous studies, according to which the application of AI as a part of systemic processes enhances its efficiency [23,57]. Consequently, the results of the present research underscore that companies must not only adopt AI but also incorporate it into their organizations’ programs.

The moderating impact of digital competence was also supported in the present study, regarding the link between AI interventions and operational efficiency in H4 and between AI interventions and patient safety in H5. These findings stress the significance of digital competencies among the staff working in the sphere of healthcare, since a higher level of competency boosts the AI solutions utilization. This is in line with past work revealing that the effectiveness of applied AI strategies depends on the client’s comprehension of and capacity to employ such tools [22]. GCC countries especially are at very different digital maturity levels, where appropriate training and development for digital competencies can greatly improve the effectiveness of applied AI initiatives. Through these relationships, this study meets an important research gap in regard to the processes through which AI interventions impact patient safety, especially in the GCC countries. Previous similar prior research has been conducted in the world healthcare system and, most of the times, failed to consider relative issues, like the diversity of the healthcare workforce, the digital preparedness, and the rapidly changing healthcare settings of the region.

The findings of this study reiterate the belief that AI adoption impacts patient safety and operational efficiency in a consistent manner across the hospitals under study. The uniformity of the selected hospitals ensures that the observed effects are not skewed by drastic variations in the healthcare structure or employees’ digital competence. While minor contextual differences do exist among GCC countries, the predominant similarities in AI integration policies, regulatory frameworks, and healthcare workforce composition allow for meaningful cross-country generalizations. This methodological consistency strengthens the implications of the findings for policymakers seeking to enhance AI-powered healthcare practices across the region.

The findings revealed by clinical performance metrics reinforce that AI interventions significantly enhance both subjective and objective measures of patient safety and operational efficiency. The institutional objective data confirm that the hospitals witnessed transformation as a result of AI integration. The incorporation of objective clinical metrics strengthens the reliability of the conclusions, addressing potential biases associated with self-reported data.

### Comparison of Patient Safety Indicators

A comparison of AI-powered interventions with traditional approaches of patient safety, as shown in the literature, was done to further elaborate the relative effectiveness of AI-based interventions for patient safety. The comparison focuses on key patient safety parameters, including diagnostic accuracy, reduction in treatment errors, timely intervention rates, and overall patient outcomes.

This AI intervention system performs at superior levels when examining patient safety key benchmarks when compared to traditional methods (Table 9). The diagnostic accuracy rate measures 95.2% with this new approach, which surpasses older methods that reached only 88.7–91.0%. The results show how decision support systems with predictive analytics capabilities improve accuracy with AI technology, which reduces diagnostic errors and leads to better clinical diagnosis. AI decreased medication errors to 1.8%, which outperformed previous reported figures between 3.2–5.0%. The combination of automated prescription validation together with real-time drug interaction monitoring and automatic dosage recommendations produces a lower occurrence of human medical errors during therapy execution. AI technology reached 92.4% success rate during timely interventions which exceeded conventional methods (83.3–87.5%). The combination of AI-driven real-time tracking systems and automatic warning capabilities decreases the response time to critical conditions and thereby enhances emergency medical services and shortens treatment periods. The clinical success of AI in healthcare reaches 94.7%, superior to traditional techniques which yield results between 86.5% and 90.1%. The combination of correct clinical diagnostics with reduced treatment errors along with faster clinical actions leads to improved recovery rates and desirable outcomes. The combination of predictive analytics with automation and real-time monitoring through AI interventions improve patient safety results across healthcare services.

## 7. Theoretical and Practical Implications

The results of this research contribute to the contemporary knowledge about the safety impacts of AI interventions, operational efficiency, and digital competence by considering the specific features of the Gulf Cooperation Council (GCC) countries’ healthcare systems. This study also adds theoretical importance to the research as we find out that both the Technology Acceptance Model (TAM) and Resource-Based View (RBV) theories are applicable in the AI context in healthcare contexts. The perceived ease of use and usefulness of TAM is reflected in the moderating variable of digital competence, while the practical application of resources postulated by the RBV theory is in congruence with the mediating variable of operational efficiency. Such paradigmatic assumptions offer an adequate conceptual background for learning about the changes AI can bring to healthcare. Thus, the results of the present research contribute to the field of understanding the positive impact of AI for patient safety by elucidating the aspects of operationality and digital literacy.

Furthermore, this study provides significant administrative and policy implications for healthcare leadership and policymakers Healthcare organizations throughout the GCC need to create AI-centered digital competency programs with universities and research facilities to boost patient safety practices. Healthcare workers must obtain AI literacy certification and hospitals need to provide routine workshops that teach both physicians and nurses to trust AI-driven choices. Healthcare institutions need to use AI for diagnostic and predictive analytics before shifting to error reduction and early condition detection. Implementing standard AI-based electronic health records (EHRs) will create optimized data exchange capabilities between all GCC hospitals. AI patient monitoring systems must assist chronic disease care by minimizing Emergency Department staffing needs. The implementation of AI governance frameworks should enforce guidelines for maintaining ethical standards together with data security measures and compliance protocols. AI ethics committees should exist in hospitals for overseeing decision systems, while data protection and AI reliability tests need periodic audits. Hospitals need to make essential investments in operational efficiency AI-based tools. The combination of AI drug management systems decreases prescriptions mistakes and AI administrative automation optimizes hospital workforce management and operating procedures. Through smart scheduling technology, hospitals should both maximize their resources and decrease patient waiting periods across the system. AI-based public health programs should address regional health problems, such as diabetes and cardiovascular conditions. Predicative analytical tools help to determine populations under risk, while AI-enabled telehealth options should provide treatment availability to underdeveloped geographical regions. GCC healthcare organizations can create an AI-driven healthcare systems oriented to local requirements by implementing the presented measures to build digital competence and optimize AI integration while raising patient safety levels.

A methodological limitation of this study is considering the GCC region as a homogeneous sample despite being distinct countries. Although there are obvious similarities among healthcare systems across the GCC region, accounting for differences will give more generalizable findings. Although the findings of this study provide valuable insights into the overall influence of AI-powered interventions in healthcare delivery across the region, future research should explore country-specific differences to come up with more targeted recommendations for the policymakers.

## 8. Conclusions

Altogether, the current paper presents strong empirical support for the change-enhancing potential of AI interventions in boosting patient safety in the healthcare organizations in the GCC. The comparative analysis revealed that the AI-supported approach shows greater diagnostic accuracy by 5.7% compared to prior studies, along with a decreased medication error rate by 1.4% and quicker intervention rate at 4.9%. The findings demonstrate why AI technologies need to be implemented because they improve operational efficiency as well as enhancing the digital skill sets of healthcare professionals. Thus, the results of the present study can enrich the literature focused on the analysis of the relationship between the application of advanced technologies and better health outcomes, by shedding light on mediating and moderating factors, such as operational efficiency and digital competence. AI interventions were also found to have primary and secondary effects on increasing patient safety by coordinating work processes, decreasing preventable mistakes, and coordinating timely actions, and it was learnt that digital competence leveraged these effects by increasing the efficient implementation and use of AI. This study also fills important research gaps by providing regional knowledge and adapting and applying existing theories such as TAM, RBV, and Systems Theory into the healthcare environment. It also has a policy-suggested action plan for promoting the digital skills, enhancing the support systems, and defining the roles of AI for every region. These observations are a reminder of how essential it is to use AI technologies purposefully to design and implement safer and more efficient healthcare systems—more of a call to action that charts the course to unlock AI’s potential and bring about the kind of systemic improvements detailed in this report.

## Figures and Tables

**Table 1:** Demographic Information of Respondents.

Demographic Variable	Category	Frequency (n)	Percentage (%)
Gender	Male	180	60%
	Female	120	40%
Age	20–30 years	100	33.3%
	31–40 years	140	46.7%
	41–50 years	50	16.7%
	Above 50 years	10	3.3%
Role	Physicians	120	40%
	Nurses	100	33.3%
	Administrative Staff	80	26.7%
Nationality	Local	93	31%
	Expatriates:		
	South Asia	72	24%
	Southeast Asia	51	17%
	Middle East	38	12.7%
	Western Countries	29	9.6%
	Others	17	5.7%
Experience in Healthcare	Less than 5 years	60	20%
	5–10 years	120	40%
	11–20 years	80	26.7%
	More than 20 years	40	13.3%

**Table 2:** Reliability and Convergent Validity.

Construct	Cronbach’s Alpha	Composite Reliability (CR)	Average Variance Extracted (AVE)
AI Interventions	0.82	0.87	0.62
Operational Efficiency	0.85	0.89	0.68
Digital Competence	0.81	0.86	0.60
Patient Safety	0.83	0.88	0.65

**Table 3:** Discriminant Validity (Fornell–Larcker Criterion).

Construct	AI Interventions	Operational Efficiency	Digital Competence	Patient Safety
AI Interventions	0.79	0.65	0.60	0.63
Operational Efficiency	0.65	0.82	0.59	0.68
Digital Competence	0.60	0.59	0.77	0.61
Patient Safety	0.63	0.68	0.61	0.81

**Table 4:** Discriminant Validity (HTMT).

Constructs	AI Interventions	Operational Efficiency	Digital Competence	Patient Safety
AI Interventions	-			
Operational Efficiency	0.72	-		
Digital Competence	0.68	0.65		
Patient Safety	0.70	0.74	0.67	-

**Table 5:** Variance Inflation Factor (VIF).

Construct	VIF
AI Interventions	2.10
Operational Efficiency	2.25
Digital Competence	1.95

**Table 6:** Model Fit Indicators.

Indicator	Value	Threshold	Result
SRMR	0.061	<0.08	Good Fit
R^2^ (Operational Efficiency)	0.41	>0.26	Substantial
R^2^ (Patient Safety)	0.46	>0.26	Substantial

**Table 7:** Path Coefficients and Hypothesis Testing.

Path	Beta Coefficient	T-Statistic	*p*-Value	Hypothesis Result
AI Interventions → Operational Efficiency	0.48	7.89	0.001	Supported
Operational Efficiency → Patient Safety	0.52	8.34	0.001	Supported
AI Interventions → Patient Safety	0.34	6.12	0.002	Supported
Digital Competence → Operational Efficiency	0.39	5.76	0.001	Supported
Digital Competence → Patient Safety	0.19	3.98	0.002	Supported

**Table 8:** Clinical Performance Metrics.

Hospital	Medication Error Rate Reduction	Patient Wait Time Reduction	Diagnostic Accuracy Improvement	AI Usage Frequency
Hospital 1	4.2% to 1.7%	55 min to 38 min	88.9% to 95.0%	70% increase
Hospital 2	5.0% to 2.0%	50 min to 35 min	89.3% to 94.8%	65% increase
Hospital 3	4.8% to 1.5%	49 min to 36 min	87.5% to 94.5%	72% increase
Hospital 4	4.6% to 1.9%	54 min to 39 min	90.0% to 96.0%	68% increase
Hospital 5	4.4% to 1.8%	53 min to 37 min	89.7% to 95.5%	74% increase

**Table 9:** Comparative Analysis of Patient Safety Parameters.

Parameter	AI-Powered Approach	Previous Work A [14]	Previous Work B [9]	Previous Work C [4]
Diagnostic Accuracy (%)	95.2	89.5	91.0	88.7
Medication Errors (%)	1.8	4.5	3.2	5.0
Timely Intervention (%)	92.4	85.0	87.5	83.3
Patient Outcomes (%)	94.7	88.2	90.1	86.5

## Data Availability

The original contributions presented in this study are included in the article. Further inquiries can be directed to the corresponding author(s).

## Appendix A. Questionnaire

Section 1: Demographic Information 1. **Gender:** ☐ Male ☐ Female ☐ Other 2. **Age:** ☐ 20–30 years ☐ 31–40 years ☐ 41–50 years ☐ Above 50 years 3. **Occupation:** ☐ Physician ☐ Nurse ☐ Administrative Staff 4. **Nationality:** ☐ Local ☐ Expatriate (if yes select region of your origin below) ☐ South Asia ☐ Southeast Asia ☐ Middle East ☐ Western Countries ☐ Others 5. **Years of Experience in Healthcare:** ☐ Less than 5 years ☐ 5–10 years ☐ 11–20 years ☐ More than 20 years
*(5-point Likert scale, 1 = Strongly Disagree, 5 Strongly Agree)*
Section 2: AI Interventions 1. The AI system in our hospital provides real-time alerts for critical patient conditions. 2. Healthcare professionals often use AI tools to support clinical decision-making. 3. The hospital utilizes predictive analytics for patient diagnosis 4. AI-powered decision support tools usually used in treatment recommendations. 5. The hospital uses AI for real-time patient monitoring and early warning alerts. 6. The hospital uses AI-driven technologies to carry out administrative tasks in hospital operations. 7. The hospital uses AI-based technologies for resource allocation and staffing in our hospital Section 3: Operational Efficiency 8. The system effectively assists in reducing task duplication. 9. The system contributes to faster decision-making in the process of patient care. 10. Operational bottlenecks are usually addressed with AI interventions. 11. The workflow digitalization minimizes delays in patient treatment. 12. The integration of AI reduces administrative workload and improves process efficiency. Section 4: Digital Competence 13. I can use the digital devices in our hospital without difficulty. 14. I can organize and store digital information efficiently. 15. I can adapt to new digital tools and technologies quickly. 16. I feel confident using AI-powered healthcare applications. 17. I have received sufficient training to effectively use AI-based systems. 18. I can troubleshoot basic technical problems when using AI systems. 19. I can integrate AI tools into my daily workflow without difficulty. 20. I am comfortable interpreting AI-generated recommendations for patient care. 21. I can critically evaluate the reliability of AI-generated clinical data. 22. I actively seek training or resources to improve my digital skills in healthcare. 23. I can navigate electronic health records (EHR) and AI-assisted documentation efficiently. 24. I understand how AI-powered decision-support systems influence patient outcomes. Section 5: Patient Safety 25. Healthcare professionals actively engage in activities to improve patient safety. 26. Hospital management provides a work climate that promotes patient safety. 27. AI-assisted decision-making reduces the chances of diagnostic errors. 28. Staff members feel comfortable speaking up if they see something that may negatively affect patient care. 29. AI-powered monitoring systems help prevent medical errors and adverse events. 30. AI integration improves communication and coordination among healthcare teams
